# Study of the Leaching of Low-Molecular-Weight Molecules from Wood-Reinforced Unsaturated Polyester Composites Using UV-Vis Spectroscopy and Gas Chromatography–Mass Spectrometry

**DOI:** 10.3390/ma19173800

**Published:** 2026-09-07

**Authors:** Vivian Müller, Felix Behrendt, Christian Dreyer

**Affiliations:** 1 Fraunhofer Institute of Applied Polymer Research IAP, Research Division Polymer Composites PYCO, Schmiedestraße 5, 15745 Wildau, Germany; christian.dreyer@th-wildau.de; 2 Department Fiber Composite Material Technologies, Technical University of Applied Sciences Wildau, Hochschulring 1, 15745 Wildau, Germany

**Keywords:** wood composites, wood preservatives, leaching, recycling, sustainability

## Abstract

**Highlights:**

- The leaching of small molecules from wood composites into water was studied by UV-Vis spectroscopy and GC-MS.
- Wood composites effectively encapsulated nonpolar compounds and retained polar compounds to a large degree (>99%).
- Using contaminated wood for wood composites is a first step to expand the lifetime of preservative-treated wood.

**Abstract:**

Wood is a sustainable and renewable resource whose various uses include heating and manufacturing of various components for different sectors. The ever-increasing global demand for wood necessitates expanding the lifetime of wood products by reusing or recycling the wood into new products, for example in wood composites. To make the case for using preservative-treated wood to produce composites, this study investigates the capability of an unsaturated polyester matrix to encapsulate low-molecular-weight molecules. Ultraviolet-visible (UV-Vis) spectroscopy and gas chromatography–mass spectrometry (GC-MS) were used to study the leaching behaviour from wood composites upon storage in water. In a first step, the wood used for the wood composites was treated with UV-active model compounds (uranine and Nile red), combined with resin to make wood composites, and their leaching into water was determined by UV-vis spectroscopy. In the GC-MS study, a similar experimental setup was used to study the leaching of wood preservatives (tebuconazole, propiconazole, and permethrin) over time from wood composites. The leaching of the wood preservatives from the wood composites upon water storage was assessed and quantified using GC-MS. The study found that the majority of the studied compounds only leach to a very small extent from the wood composites. Possible reasons for variations in leaching behaviour are discussed. These findings are an important first step in the endeavour of reusing contaminated wood in wood composites.

## 1. Introduction

Wood is one of the most versatile and widely utilised natural resources worldwide, fulfilling essential functions in energy generation, building construction, furniture manufacturing, and a growing number of industrial applications [1,2]. As a renewable, carbon-sequestering material, it has attracted increasing scientific and political attention as a sustainable potential substitute for carbon-intensive, non-renewable materials, such as concrete, steel, and petroleum-based plastics [3,4].

Materials that combine wood fibres or wood flour with thermoplastic (i.e., wood–plastic composites, WPC) or thermoset polymer matrices, such as unsaturated polyesters (here wood composites), have emerged as particularly promising classes of materials with high bio-derived content. Wood composites offer a favourable combination of mechanical performance, dimensional stability, and reduced environmental footprint compared with purely synthetic alternatives, and their global market has grown steadily over the past two decades [5,6].

Global demand for virgin wood continues to accelerate at an unprecedented pace. According to the Food and Agriculture Organization of the United Nations (FAO), worldwide roundwood production reached a record high of approximately 4.0 billion cubic meters in 2022, and the production of round wood equivalents is projected to increase by 37% between 2020 and 2050 [7]. While the substitution of fossil-based materials with wood products is broadly considered to be environmentally beneficial [3,8], the increasing demand raises serious concerns regarding forest-resource sustainability, biodiversity loss, and rising commodity prices [9,10]. These concerns are compounded by the inefficiency of current end-of-life wood management: a substantial share of post-consumer wood is incinerated for energy recovery rather than being materially recycled [11,12].

A central barrier to the circular use of wood lies in the widespread application of chemical treatments, most notably wood preservatives. Even though more environmentally benign wood preservation techniques are being developed [13], timber destined for outdoor, structural, or ground-contact applications is routinely treated with biocidal compounds, such as copper azole, propiconazole, tebuconazole, or 3-iodo-2-propynyl butylcarbamate (IPBC), in order to prevent fungal decay and insect attack [14,15]. In the European Union, the end-of-life management of chemically treated wood is strictly regulated. Under the Waste Framework Directive [16] and the Biocidal Products Regulation [17], treated wood is classified in ways that largely restrict its disposal pathway to incineration in licensed facilities, particularly when hazardous substances are present [16]. The principal rationale underlying these restrictions is that wood products using treated wood may leach wood preservatives or their degradation products when they are exposed to water or weathering, posing potential risks to human health and the environment [18,19]. As a consequence, the wood fibre currently used in WPCs and wood composites is predominantly virgin, untreated material, which further intensifies pressure on primary forest resources and runs counter to circular-economy objectives [12,20].

The incorporation of treated, post-consumer wood into wood composites could yield substantial environmental and economic benefits, provided that the migration of preservative compounds from the composite matrix can be effectively restricted [21,22]. Several studies have demonstrated that the polymer matrix can act as a partial barrier to the release of embedded substances, yet the degree of encapsulation depends on a complex interplay of factors, including matrix type, filler loading, compound polarity, molecular size, and composite porosity [23,24,25]. Crucially, we have not found any studies investigating the influence of resin composition on the leaching behaviour of small organic molecules from wood composites, and most studies of leaching from wood composites and the corresponding analytical methods used for quantification focus on inorganic molecules [26,27,28]. As a consequence, this knowledge gap hampers the development of wood composites as safe second-life products incorporating treated wood.

The present study aims to make a first step towards enabling the incorporation of preservative-treated wood in wood composites by studying whether and to what extent a polymeric resin can encapsulate small molecules. In the first stage, the migration behaviour of small chemical compounds from wood composites was investigated by ultraviolet-visible (UV-Vis) spectroscopy using two model-compound dyes of different water solubility, while varying the resin composition to identify matrix formulations that minimise leaching. In the second stage, the most promising resin composition was employed to study the leaching of three representative wood preservatives (tebuconazole, propiconazole, and permethrin) from the corresponding composites, with quantification by gas chromatography–mass spectrometry (GC-MS). Specifically, the objectives of this work were to (a) study the migration behaviour of small chemical compounds from wood composites with different chemical properties, (b) evaluate the influence of resin composition on migration behaviour, and (c) develop an analytical method capable of assessing the leaching of model organic wood preservatives from wood composites. 

## 2. Materials and Methods

### 2.1. Materials 

The following chemicals were used: Nile red (Carl Roth GmbH & Co KG, Germany) and uranine (Carl Roth GmbH & Co KG, Germany) as dyes, Polylite 31696-55 (20 kg, Polynt, Germany) as resin, divinylbenzene (DVB, Isomer mix, 1 L, Merck), 4-acryloylmorpholine (ACMO, 500 mL, Rahn, Germany), and styrene (S, 2.5 L, VWR) as reactive diluents, Peroxan PB (50 mL, Pergan) as an initiator (I), MD700 (M1, Solvay) and BYK3505 (M2, BYK, Germany) as surface-active additives, Korasit NG10 (Holz Possling, Germany) as a wood preservative, tebuconazole (analytical standard, 250 mg, Pestanal), propiconazole (analytical standard, 250 mg, Ehrenstorfer), and permethrin (analytical standard, 100 µg/mL in cyclohexane, 1 mL, Ehrenstorfer) as active chemical ingredients of the chosen wood preservative, and deionised water (in-house), acetonitrile (MeCN, LC grade, 2.5 L, Supelco), and ethanol (99%, Caldic).

### 2.2. UV-Vis Study

#### 2.2.1. Sample Preparation and Leaching

Spruce wood (*Picea abies*), a commonly used wood in Europe, was milled in a ring chipper (CSK 350NI, Condux, Netzsch, Hanau, Germany) and fractionated (2–4 mm; 0.63–2 mm; <0.63 mm) in a sieve tower (Retsch VS 1000, Retsch GmbH, Haan, Germany) at 50 Hz for 2 min. The wood millings were dried at 80 °C in the oven for an hour prior to sample preparation. For the samples of the UV-Vis study, wood millings (fraction size 0.63–2 mm) were pretreated with dye solutions. The dyes uranine and Nile red were used. For both dyes, a solution (concentration 0.1 g/L) was prepared by dissolving uranine in deionised water and Nile red in ethanol. Subsequently, 4 mL of the solution were used per g of wood millings. The mixtures were stirred in a round-bottomed flask for one hour by continuous rotation of the flask on a rotary evaporator to ensure a uniform dye uptake (no vacuum was used). After soaking, the wood chips were dried at room temperature and then at 60 °C to remove solvent residues and to ensure a uniform distribution of the dye within the carrier material. Per wood composite sample, pre-treated millings (7.5 g) were combined with resin (3.21 g). Different resin mixtures were prepared by combining a resin, reactive diluent, initiator, and, if applicable, a surface-active additive and homogenising the mixture in a speed mixer (SpeedMixer DAC 400.1 VAC-P, Hauschild, Germany) at 2000 rpm for five minutes. The compositions of the resin mixtures are summarised in Table 1.

**Figure A1:**
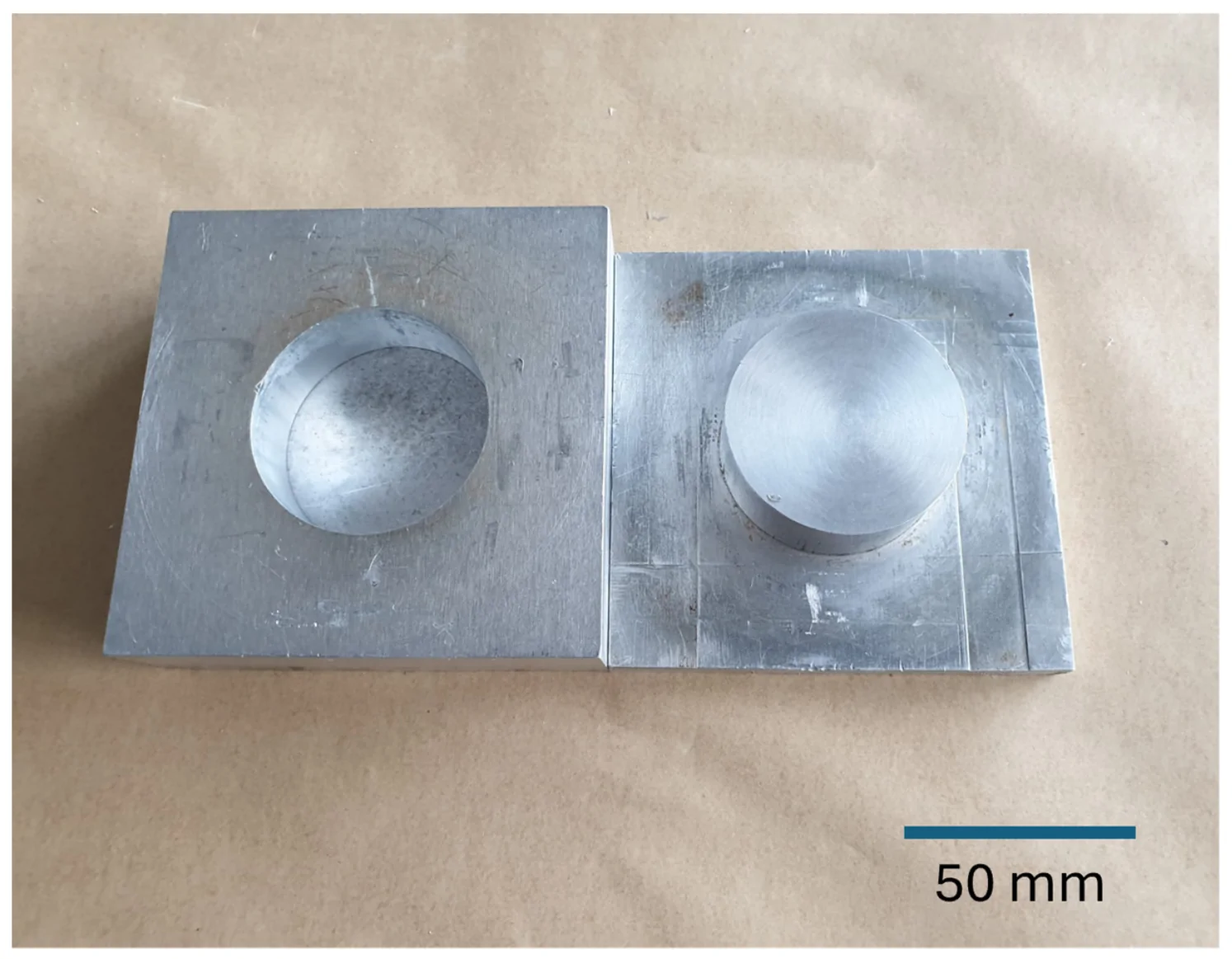
Mould used for the preparation of the pucks with an inner diameter of 50 mm.

**Figure A2:**
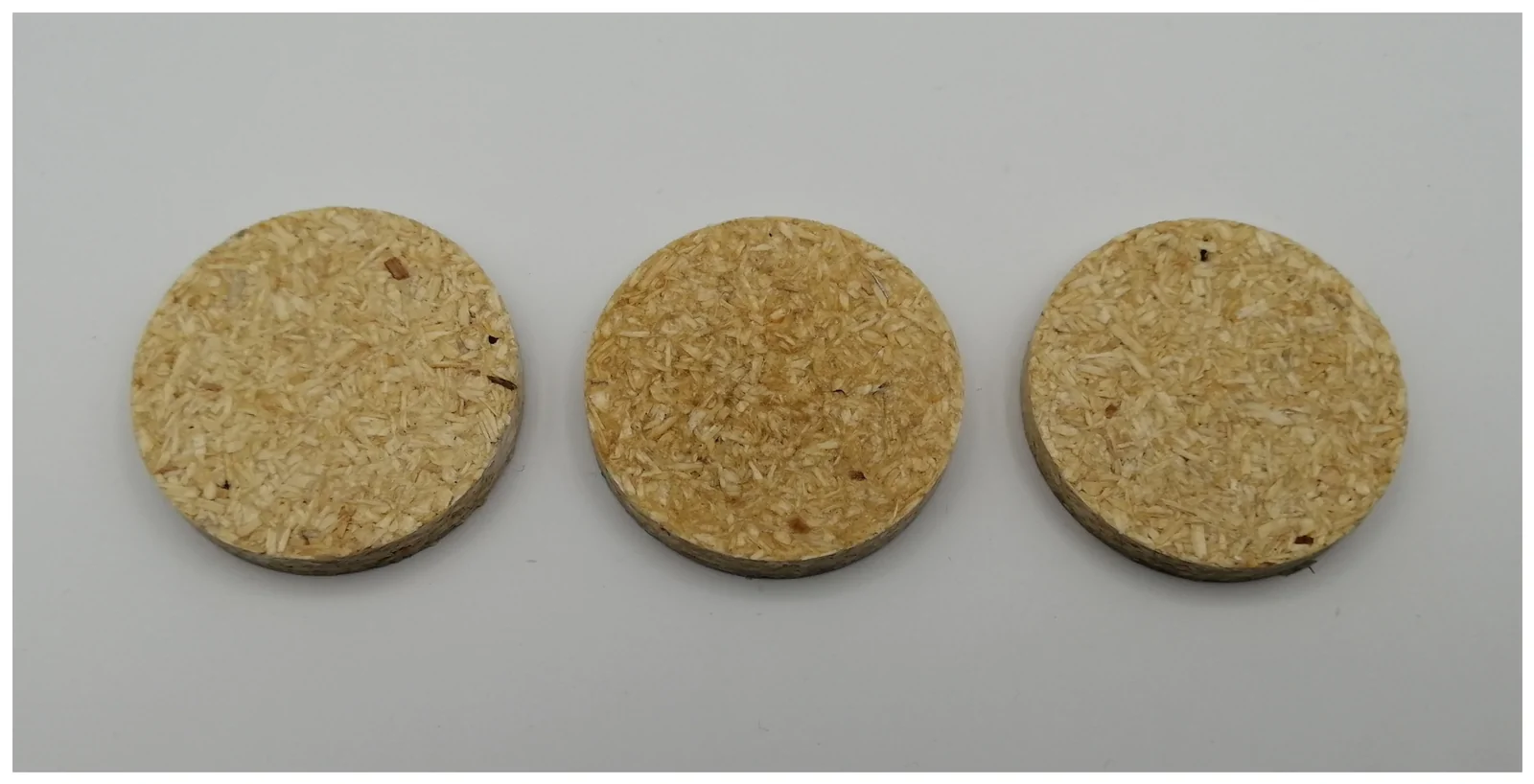
Picture of three wood composite samples with a resin content of 30%.

The millings were mixed with a resin mixture in a speed mixer (SpeedMixer DAC 400.1 VAC-P, Hauschild, Germany) at 2000 rpm for five minutes. After mixing of the millings with resin, the material was filled into a mould with an inner diameter of 50 mm (Figure A1) and pressed to a defined thickness of 4 mm using a hand press (Rucks, Germany) at 70 kN and 130 °C for 30 min. The samples (50 mm diameter, 4 mm thick) were subsequently tempered for two hours at 130 °C in an oven. A photo of the samples is shown in Figure A2.

The samples were immersed in deionised water (100 mL) and kept in the dark at 30 °C without stirring. Then, 1 mL of water was withdrawn for the UV-Vis measurements after 11 days.

#### 2.2.2. Instrumentation

The UV-Vis spectra were measured in quartz cuvettes (2 mL, path length 1 cm, Macherey–Nagel, Düren, Germany) using a UV-Vis spectrometer (UV5, Mettler-Toledo, Gießen, Germany).

#### 2.2.3. Data Treatment

**Figure A3:**
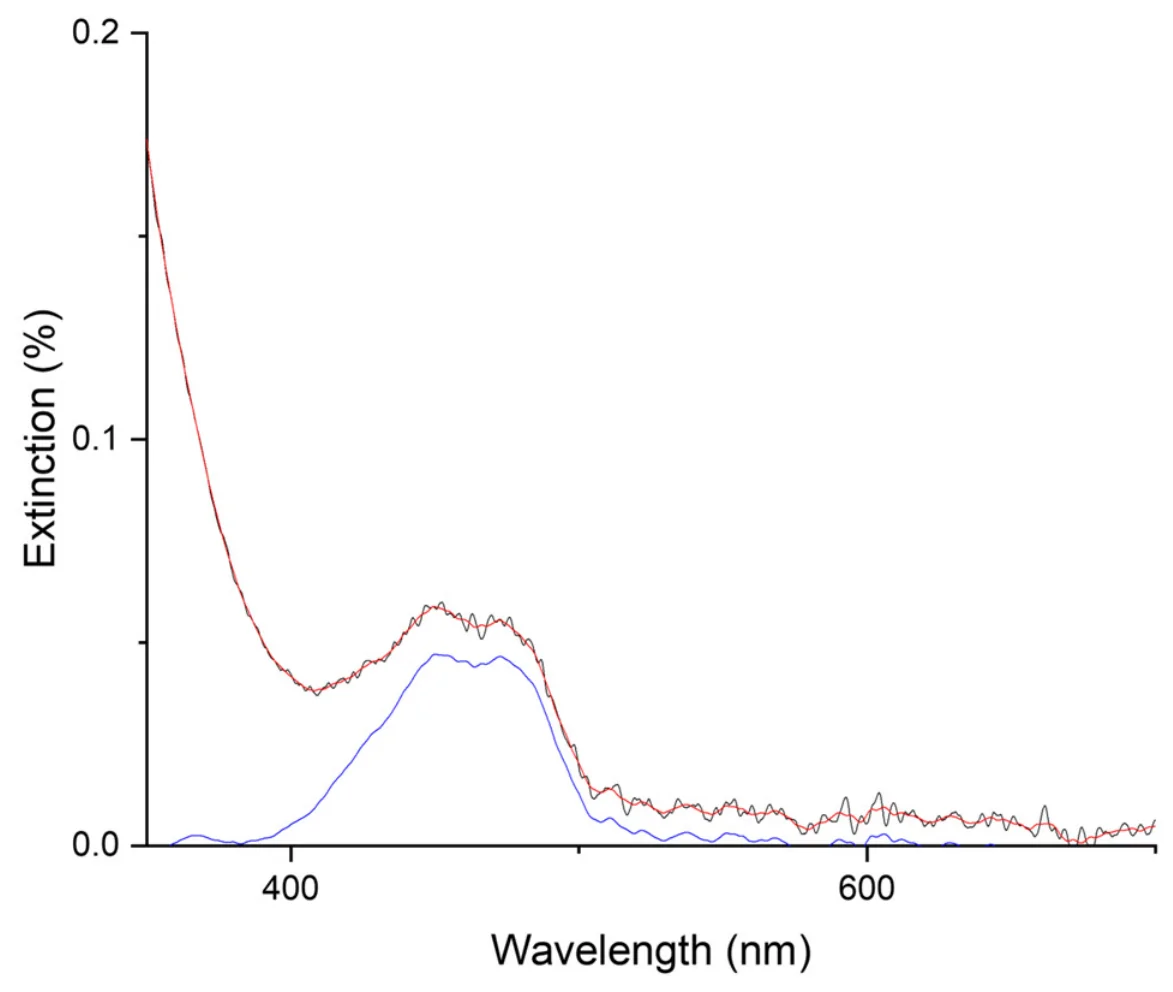
Treatment of the UV-Vis spectroscopy data. The raw data are shown in black, after smoothing with the Savitzky–Golay algorithm in red, and after background subtraction in blue.

**Figure A4:**
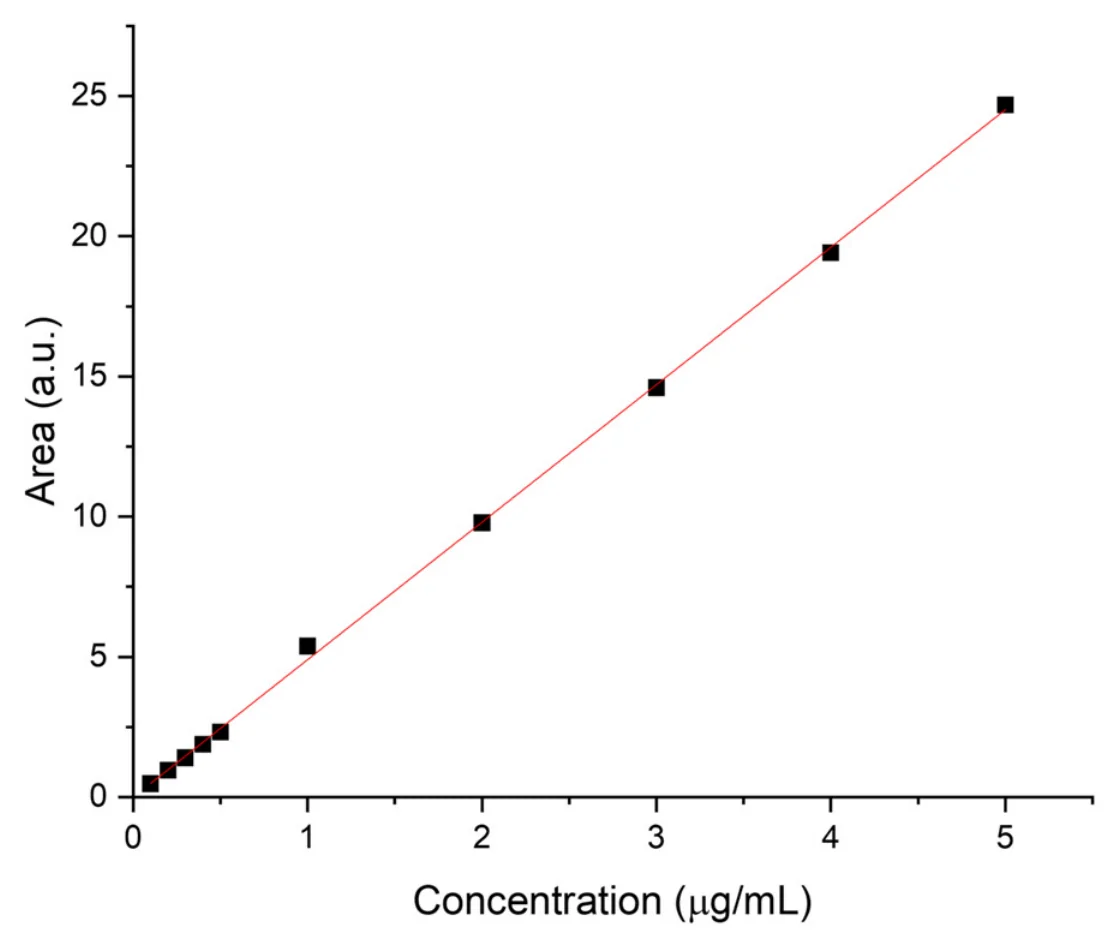
Calibration curve of uranine in water obtained by integrating the signal between 390 and 510 nm.

The data treatment was performed in Origin (OriginPro, Version 2021 (64-bit version), OriginLab Corporation, Northampton, MA, USA). To obtain a better signal-to-noise ratio, Savitzky–Golay smoothing was applied to all spectra. For each resin matrix, a triplicate determination was carried out to achieve the highest possible statistical reliability. Background subtraction was performed because the signal peak to be evaluated appeared only as a shoulder on a clearly larger background signal. To address this problem, the background in the region around the dye peak was first modelled using exponential peak fitting. The goal was to reproduce the course of the overlapping larger peak as accurately as possible. Subsequently, this modelled background was subtracted from the original spectral data, allowing the UV-Vis signal of the dye to be isolated. For a visualisation of the modification of the data, see Figure A3. After background correction, the relevant peak areas (between 390 and 510 nm) could be integrated using the calibration curve (calibration curve for uranine is shown in Figure A4). This integration enabled the quantitative evaluation of the dye concentration in the respective sample solutions.

### 2.3. Gas Chromatography–Mass Spectrometry Study

#### 2.3.1. Sample Preparation for GC-MS Measurements and Leaching

For the manufacturing of the samples, wood chips with a fraction size of 0.63–2 mm were used and treated with the wood preservative Korasit NG10, which contains the active ingredients tebuconazole, propiconazole, and permethrin. Pre-dried wood was mixed with Korasit NG10 in a 1:0.75 ratio and homogenised in a 500 mL round-bottomed flask on a rotary evaporator (no vacuum). After the wood preservative was completely absorbed by the wood chips, the material was mixed with resin formulation 1 (see Table 1). Depending on the sample, 30% or 40% resin was used and the corresponding mixture was filled into the mould (50 mm inner diameter, thickness 4 mm). After pressing, the pucks were post-cured in the oven at 130 °C for two hours. To induce leaching, the pucks were placed in 50 mL of water, stored in dark conditions at room temperature without stirring, and sampled (1 mL) after 1 day, 6 days, and 26 days.

For the resin-free reference samples, two distinct approaches were chosen. In the first approach, three wood pucks (WPs; diameter 50 mm, thickness 4 mm) were milled from spruce wood (*Picea abies*) and soaked with Korasit NG10 (ratio of wood:wood preservative of 1.5:1). Subsequently, they were placed in 50 mL of deionised water. In the second approach for reference samples, wood millings (fraction size 0.63–2 mm) were soaked in wood preservative with a wood:wood preservative ratio of 1:0.75. The millings were placed in 100 mL of deionised water (the higher amount was necessary to completely immerse the millings). The resulting theoretical wood preservative concentrations are summarised in Table 2.

#### 2.3.2. Instrumentation and GC-MS Measurements

The GC-MS measurements were conducted on a Shimadzu GCMS-QP2020NX instrument (software version GCMSsolution version 4.53SP1), using a Shimadzu Rtx-5MS column, a helium flow rate of 32.4 mL, and electron ionisation (EI) mode. Each sample was analysed three times by GC-MS, with 8 µL of the sample injected each time, using a split ratio of 20. The multiple injections are intended to compensate for potential fluctuations of the GC or MS instrument and to improve the reproducibility and accuracy of the analysis. The temperature program started at 100 °C, increasing at a rate of 40 K/min to 180 °C, and subsequently at a rate of 20 K/min to 310 °C. The final temperature was held for 0.8 min. The scan range was set to 35.00–280.00 *m*/*z* with a scanning speed of 909 and sampling time of 0.3 s. In addition, single-ion monitoring (SIM) was carried out for the following *m*/*z*: 259.00, 173.00, and 69.00 (characteristic for propiconazole), 250.00, 125.00, and 70.00 (characteristic for tebuconazole), and 183.00, 165.00, and 163.00 (characteristic for permethrin). The *m*/*z* values in bold are the ones used for quantification.

#### 2.3.3. Calibration Curves

The data were quantified using the calibration curves. For the calibration solutions, stock solutions containing the compounds tebuconazole, propiconazole, and permethrin were prepared in acetonitrile. 

**Figure A5:**
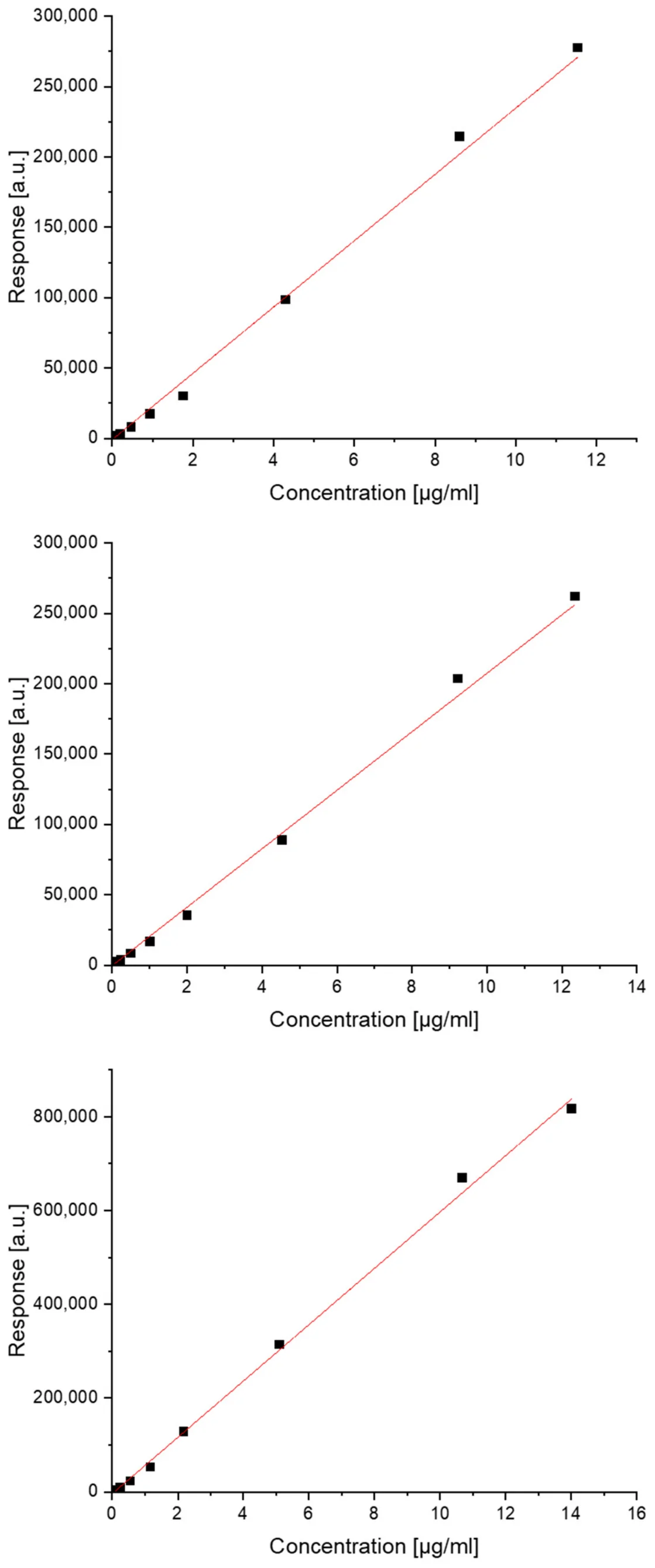
Calibration curves of tebuconazole (**top**), propiconazole (**middle**), and permethrin (**bottom**) with R^2^ values of 0.9907, 0.9931, and 0.9958, respectively.

**Figure A6:**
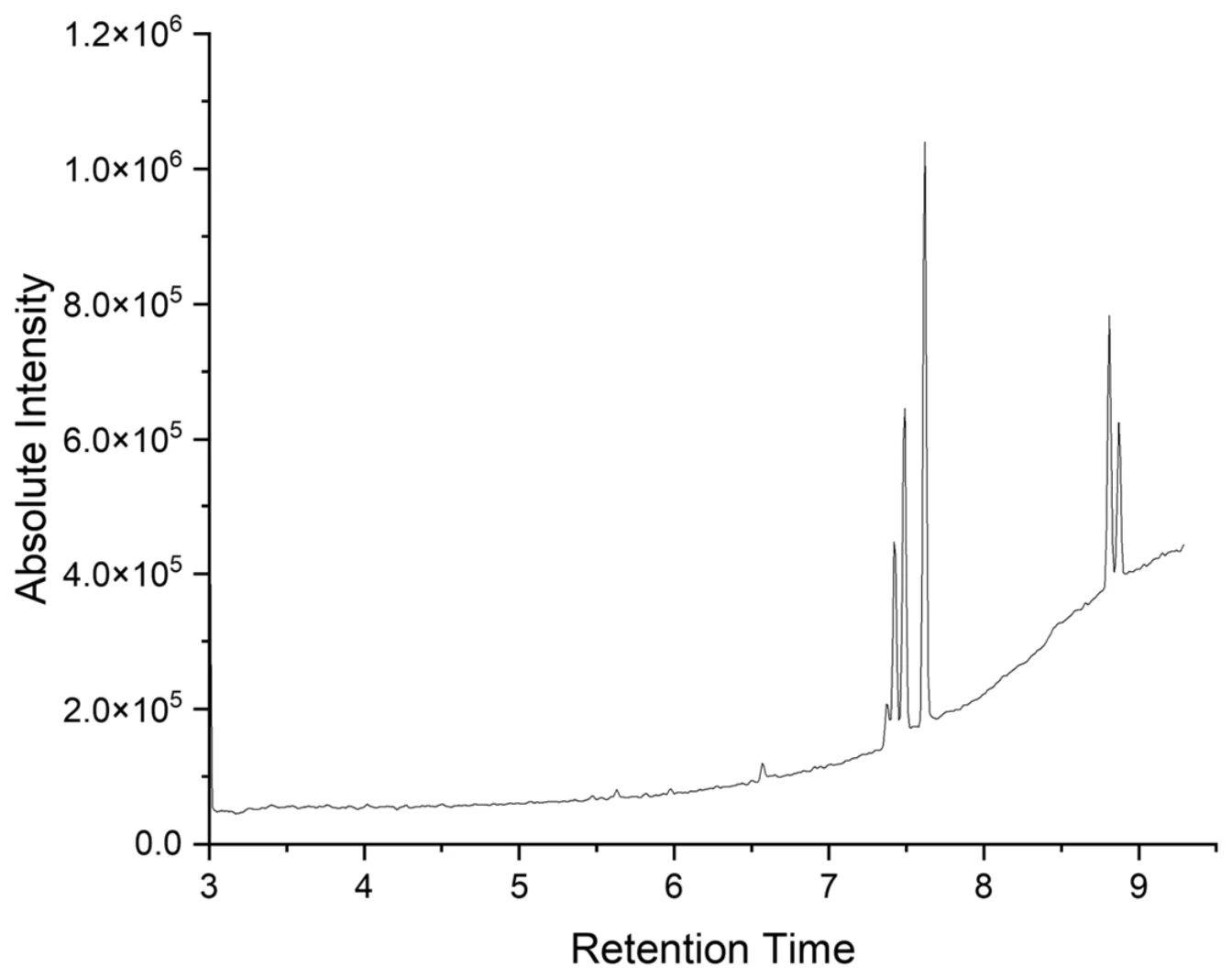
Chromatogram of a solution of propiconazole (RT: 7.4 and 7.5 min), tebuconazole (RT: 7.6 min), and permethrin (RT: 8.8 min and 8.9 min, two peaks cis/trans) with a concentration of approximately 5 µg/mL.

The calibration curves for the individual compounds had R^2^ values of 0.9907 (tebuconazole), 0.9931 (propiconazole), and 0.9958 (permethrin), thus all exceeding the generally accepted threshold value for calibration curves (>0.990). The curves are depicted in Figure A5. An example of a chromatogram of the calibration solution at a concentration of 0.1 µg/mL is shown in Figure A6. The limit of detection (LOD) and limit of quantification (LOQ) of the GC-MS method were determined from the calibration curves using the slope of the regression line (*S*) and the residual standard deviation of the response (σ):

(1) $$LOD=\;\frac{3.3\;\sigma }{S}\;\mathrm{and}\;LOQ=\;\frac{10\;\sigma }{S}$$

Only the five lowest concentration levels of the calibration curve were included in the regression used for the LOD and LOQ calculations.

The resulting LOD and LOQ values were 0.083 and 0.25 µg/mL for tebuconazole, 0.080 and 0.24 µg/mL for propiconazole, and 0.061 and 0.19 µg/mL for permethrin, respectively.

## 3. Results

### 3.1. Leaching of UV-Active Model Compounds from Wood Composites Treated with UV-Active Compounds 

To gain an understanding of whether small molecules encapsulated by the resin within a wood composite leach out when submerged in water, a first evaluation of the leaching from wood composites was carried out by UV-Vis spectroscopy. Different wood composites samples were prepared, varying the resin composition.

#### Influence of Resin Matrix Formulation

The main aim of this study was to quantify the migration of apolar and polar low-molecular-weight substances from wood composites treated with UV-active compounds in an aqueous environment under defined conditions. Two dyes were chosen as model compounds to assess the leaching of small molecules from the wood composites: uranine (a water-soluble sodium salt of fluorescein) as a water-soluble dye and Nile red as a nonpolar dye. Additionally, the resin matrix formulation was varied in order to study the effect of the modifications on the leaching behaviour. The unmodified resin (R) served as a reference or baseline to assess the effects of resin variation on the leaching behaviour. Different reactive diluents were used. Styrene (S) was employed as an apolar standard reactive diluent.

Acryloylmorpholine (ACMO) served as a highly reactive, polar diluent, while divinylbenzene (DVB) was used as a multifunctional reactive diluent with the aim of potentially achieving a higher network density in the cured resin. The influence of surface-active additives on the properties of the resin formulations was also investigated. Two different additives were tested: a bifunctional, reactive PFPE-urethane-methacrylate (M1), and an acrylate-modified silicone (M2). Both additives were used with a concentration of 0.5 mass percent, each in combination with styrene as the reactive diluent. The wood composite samples were prepared according to the procedure outlined in the Section 2. To simulate leaching, the samples were transferred into a defined volume of water and stored at a constant temperature of 30 °C for eleven days. The concentration of the dyes in the water was determined by UV-Vis spectroscopy after eleven days. In order to quantify these amounts, calibration curves were prepared for both dyes. For the uranine samples, the standards were prepared directly in water. Since Nile red has only very limited water solubility, the samples could not be dissolved directly in water. Due to the pronounced solvatochromic effect, direct measurement in an organic solvent was also not possible. Therefore, a highly concentrated solution in ethanol was first prepared and then added to the required amount of water.

**Figure 1:**
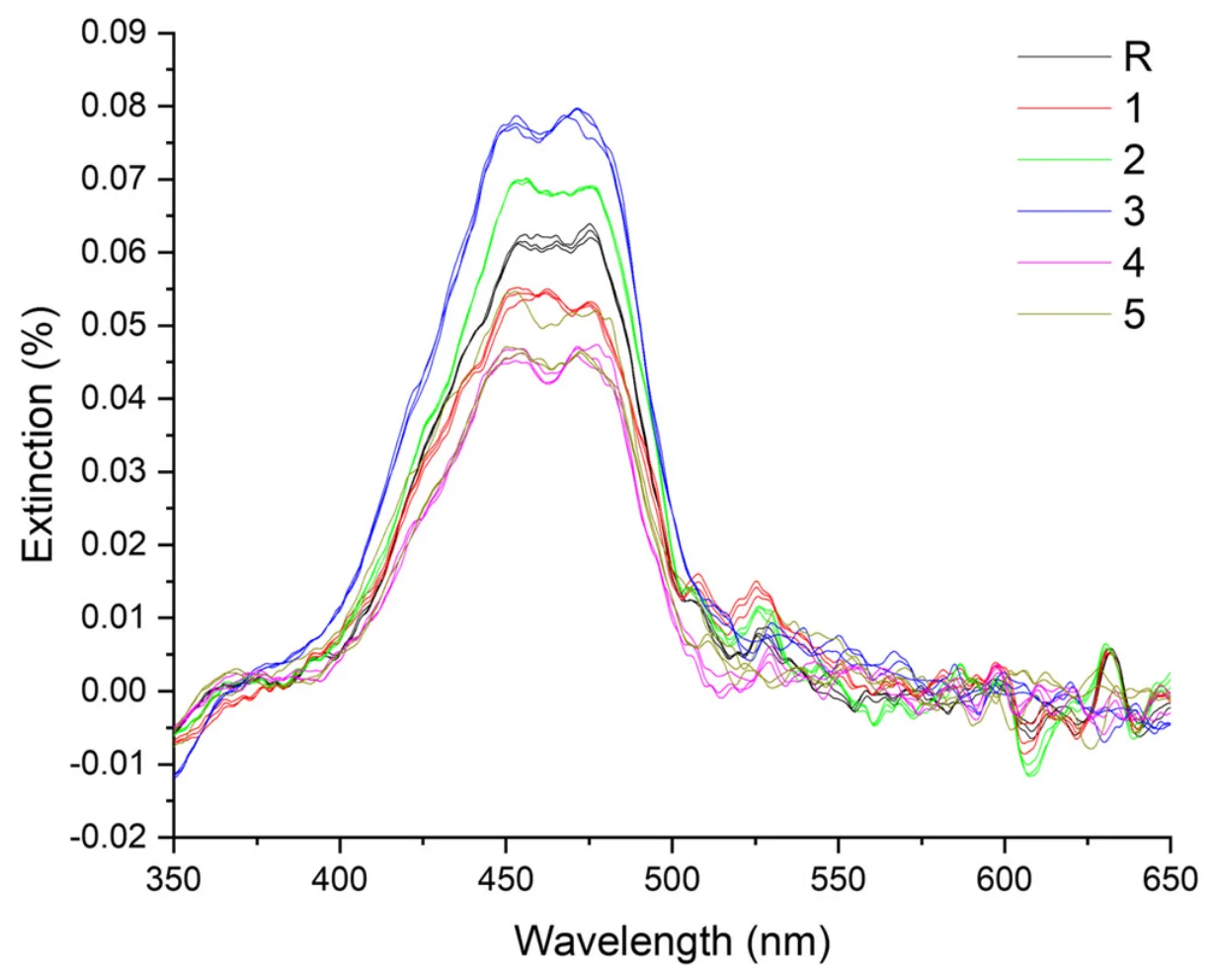
UV-Vis spectra of the solutions in which the wood composites differing in resin formulation (unmodified resin R and modified resin 1–5) and pre-treated with uranine had been stored for eleven days.

No signal for Nile red could be detected for any of the wood composite samples, which suggests that the amount of Nile red that had leached into the water was below the limit of detection for all samples. This suggests that regardless of the matrix composition, all wood composites can prevent the leaching of this substance to a high extent. On the other hand, uranine was found in the water of all uranine-containing pucks irrespective of the matrix composition; the amount, however, varied depending on matrix composition (Figure 1). 

The UV-Vis measurements indicate that the addition of ACMO or DVB (mixtures 2 and 3) leads to an increased leaching of uranine. On the other hand, the addition of styrene (mixture 1) or modifiers M1 or M2 (mixtures 4 and 5) seem to lead to reduced leaching of uranine compared to the reference, R. For the quantitative evaluation of these samples, a comprehensive data analysis of the spectra was performed (details in the Section 2). After data processing, the obtained peak areas were converted to concentrations using the calibration curves prepared beforehand (Figure A1) to determine the quantities of uranine that had leached into the water. The fraction of the dye leached from the material relative to the originally used total amount could thereby be determined. The results are summarised in Table 3.

The measured concentrations of uranine leached from the material were below 1 µg/mL for all samples. This corresponds to about 2–4% of the theoretical possible maximum value and indicates only a low release of the dye uranine from the investigated materials. Both DVB and ACMO seem to have a negative impact on the retention behaviour of the pucks. The samples diluted with styrene show a slightly improved retention behaviour compared to the reference resin. A further slight improvement of the retention behaviour was obtained with silicon-modified and PFPE-modified material. However, the use of these surface-active materials carries the risk that they could be released into the environment. Since the improvement in retention behaviour was only marginal, the difference to the samples diluted only with styrene was deemed insufficient to justify their use. Overall, the results indicate that both substances seem to be effectively encapsulated inside a wood composite. Resin formulation 1 was used to further study the encapsulation ability of the wood composites by GC-MS.

### 3.2. Study of Leaching of Wood Preservatives from Wood Composites by GC-MS

#### 3.2.1. GC-MS Study of Wood Composites

The leaching of three wood preservatives from wood composites was studied by GC-MS. The contamination of fresh and aged wood with wood preservatives has previously been studied by GC-MS [29,30]. The focus of these studies was on quantifying the wood preservative residues contained in the wood. Based on these methods, a GC-MS method was developed to determine the amounts of tebuconazole, propiconazole, and permethrin that leach out of the wood composites during water storage. The details of the GC-MS method can be found in the Section 2.

In a first step, a calibration curve was established for the wood preservative components tebuconazole, propiconazole, and permethrin. Calibration solutions with certified standards were prepared in a concentration range from 0.05 µg/mL to 15 µg/mL. Permethrin has a very low solubility in water (0.2 µg/mL at 20 °C); therefore, acetonitrile was chosen as a polar solvent rather than water. The upper limit of the calibration range (15 µg/mL) corresponds to the solubility threshold of the wood preservatives in acetonitrile, as above a concentration of 15 µg/mL solubility issues occurred. The lower limit of the concentration range corresponds to the detection limit of permethrin with the method used (Equation (1)). For further details, see the Section 2.

To simulate the leaching behaviour of heavily contaminated samples, a treatment procedure was chosen in which the resulting pucks have a wood preservative concentration well above the typically used concentrations. Pucks with a resin content of 30% and 40% were prepared in triplicate and stored in water at 30 °C. After 1 day, 6 days, and 26 days, samples were taken and subjected to GC-MS analysis.

**Figure 2:**
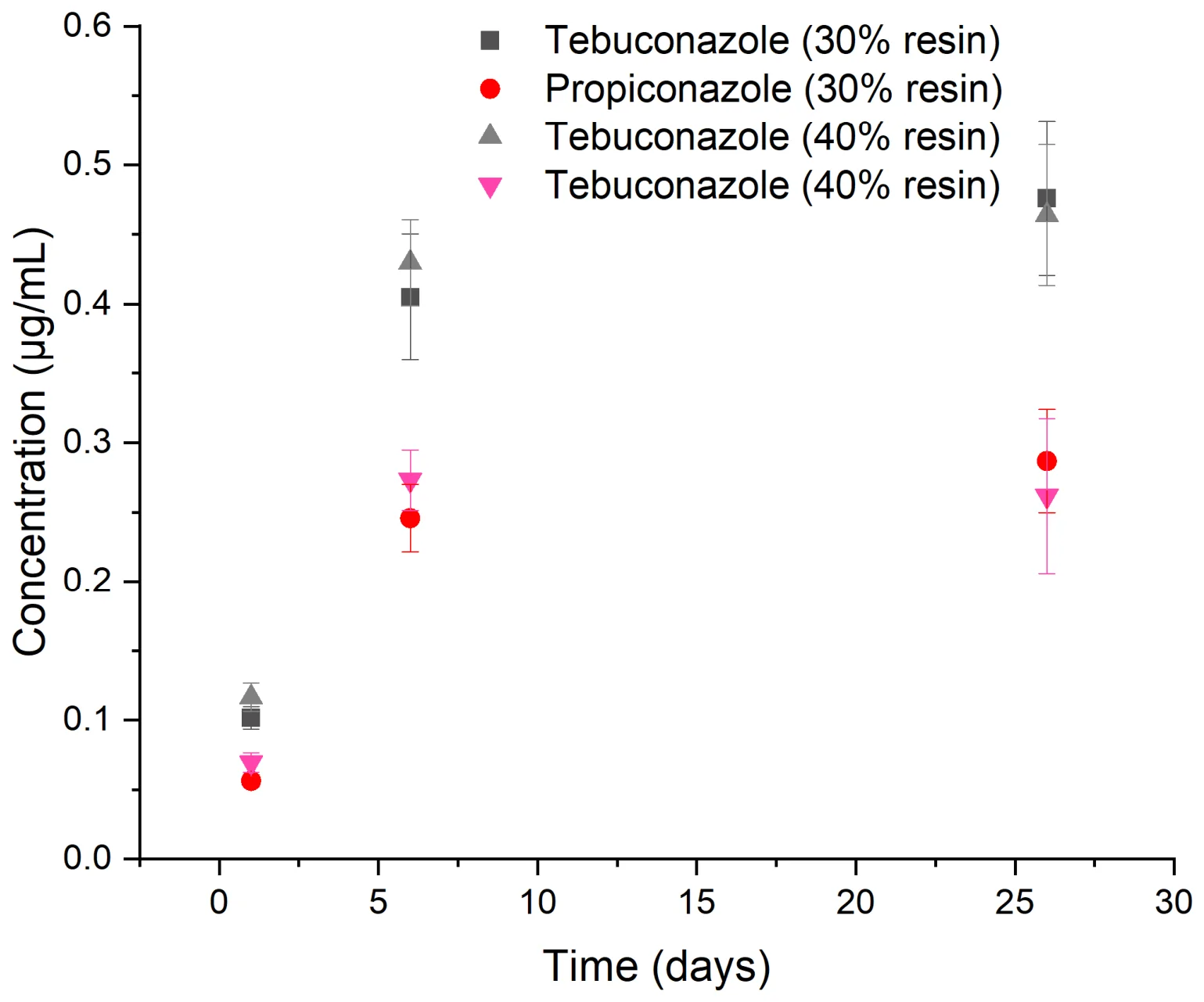
Leaching of propiconazole and tebuconazole from wood composites with different resin contents (30% resin, circles and 40% resin, triangles) into water: the concentrations of propiconazole (red and pink) and tebuconazole (black and grey) measured in the water over time, as determined by GC-MS. The sampling was done after 1 day, 6 days, and 26 days.

While both propiconazole and tebuconazole were detected by GC-MS analysis, none of the samples seemed to contain permethrin. In Figure 2, the concentrations of propiconazole and tebuconazole detected in the samples over time as a function of the resin content used are shown. Each data point represents the mean of the obtained values from three samples (each measured in triplicate), with error bars indicating the standard deviation.

The wood preservative Korasit NG10 contains 250 mg of permethrin, 150 mg of tebuconazole, and 150 mg of propiconazole per 100 g of Korasit NG10. For each puck, 7.5 g of treated wood were used, corresponding to 5.625 g of wood preservative. Thus, per puck, a maximum of 14.0625 mg of permethrin should be present. Since the pucks were immersed in 50 mL of water and 1 mL was withdrawn for each analysis, a maximum of 281 µg of permethrin is to be expected per sample. Analogously, a maximum of 169 µg/mL of tebuconazole and a maximum of 169 µg/mL of propiconazole are to be expected.

It is evident that significantly more tebuconazole than propiconazole diffuses out of the wood pucks, yet the concentrations for both substances lie in the range of 0.05–0.50 µg/mL. The resin content (30% vs. 40%) appears to have no influence on the diffusion ability of the substances, with hardly any difference between the pucks with a resin content of 30% and those with a resin content of 40%.

Permethrin could not be detected in any of the samples. This means that either no permethrin or less than 0.06 µg/mL of permethrin (corresponding to the detection limit of permethrin) diffuses from the pucks. The latter value corresponds to less than 0.01% of the originally used amount. 

The amount of tebuconazole detected after one day is approximately 0.1 µg/mL, which corresponds to 0.05% of the applied tebuconazole. Between the first (day 1) and the second sampling (day 6), the concentration of tebuconazole shows a marked increase, whereas barely any further increase can be seen between the second (day 6) and the third samplings (day 26). The maximum concentration of tebuconazole in a single sample is 0.47 µg/mL. For propiconazole, a similar trend is observed, however, less propiconazole is found in the water. The maximum concentration of propiconazole is observed in the last sample and is about 0.28 µg/mL. More than 99% of the wood preservative is, therefore, bound by the resin matrix. These results show that the encapsulation capacity of the resin matrix is very high, but also that it varies according to the substance.

#### 3.2.2. GC-MS Study of Resin-Free Reference Wood Samples

Reference samples were prepared to assess whether the retention of the compounds is due to the ability of the resin formulation to bind the components of the wood preservative. As the wood chips by themselves do not stick together, it was not possible to produce pucks without resin. Instead, two different approaches were chosen to obtain reference values. On one hand, wood pucks with a thickness and a diameter equal to the wood composites and, therefore, the same surface-to-volume ratio, albeit a lower mass, were produced. They were subsequently soaked with wood preservative before conducting the leaching experiment. On the other hand, the same amount of wood-preservative-treated wood chips that were used for the wood composite preparation served as a reference material and were also stored in water to determine the leaching. The exact procedure and the theoretical maximum amounts of the wood preservatives are listed in the Section 2. Within a few hours of placing the reference materials in water, the water in which the treated wood pucks or reference wood chips were placed turned intensely red. This effect was attributed to the colour of the wood preservative. Visually, it does not appear that a large amount of the wood preservative is bound by the wood. To verify and quantify this, the samples were subjected to GC-MS analysis.

**Figure 3:**
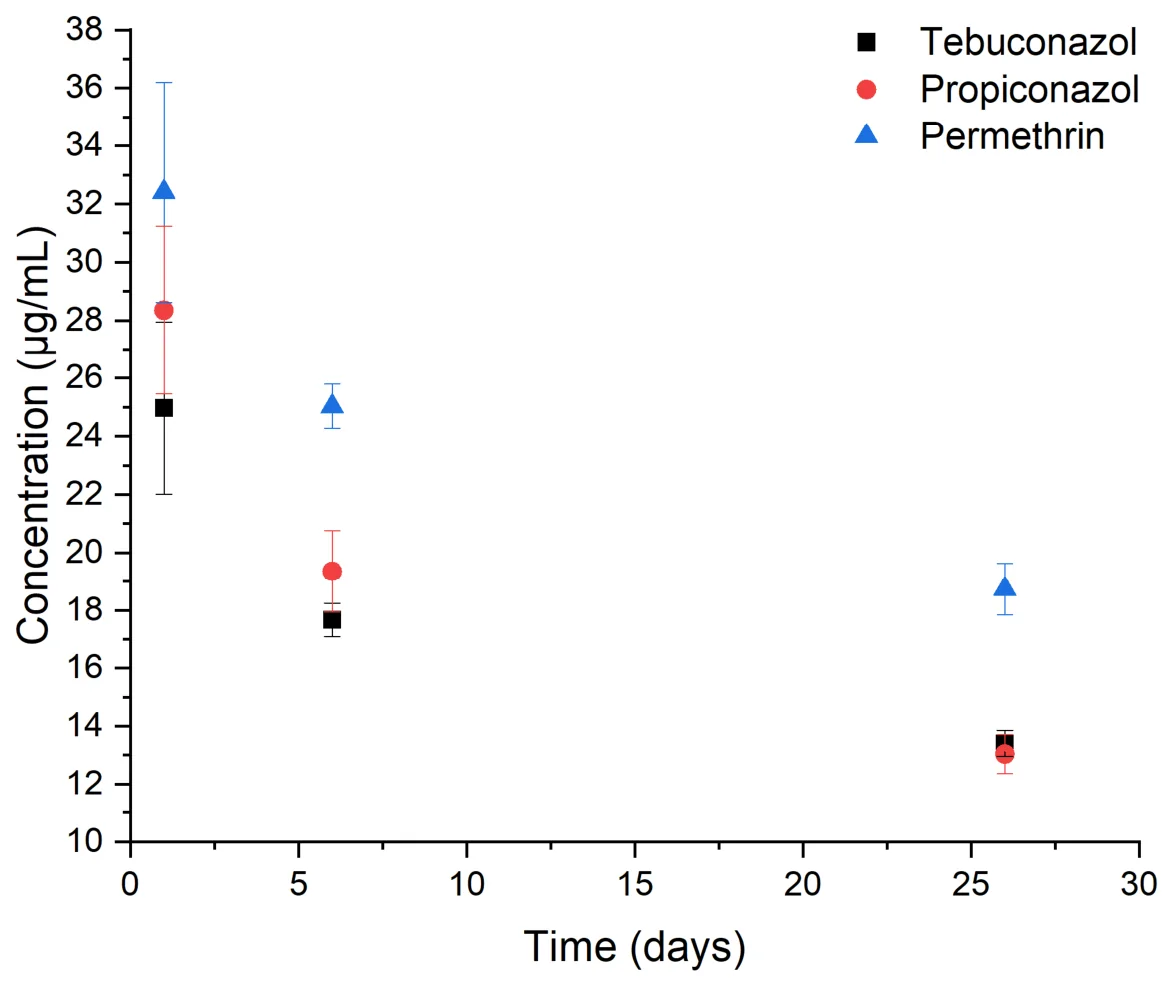
Leaching of propiconazole and tebuconazole from wood reference samples into water: the concentrations of propiconazole (red), tebuconazole (black), and permethrin (blue) measured in the water over time, as determined by GC-MS. The sampling was done after 1 day, 6 days, and 26 days.

The results show that the amounts of released propiconazole, tebuconazole, and permethrin from the reference samples are significantly higher than those from the wood composites. After one day of water storage, the concentrations in the samples lie approximately between 25 and 32 µg/mL (Figure 3). The released amounts of the individual wood preservative differed only slightly, although permethrin unexpectedly leached to a greater extent than the other two compounds. During the preparation of the calibration solutions, solubility limitations restricted the calibration range to a concentration of 15 µg/mL. Therefore, the measured concentrations should be interpreted with care, as they lie outside of the calibration range. Even so, the permethrin concentrations were considerably higher than expected, exceeding the reported solubility limit of approximately 0.2 µg/mL by far. This discrepancy between the dissolution behaviour and the concentration upon leaching may be due to a co-leaching of compounds (for instance, emulsifiers present in the formulation Korasit NG10) that could effectively increase the maximum solubility of the compounds. 

After 6 and 26 days, the concentrations of the three substances in the samples are lower. Interestingly, the concentrations of tebuconazole and propiconazole fall to 13–14 µg/mL over time, and the concentration of permethrin to 18 µg/mL. These values approach the solubility limit observed for the calibration solutions. We, therefore, assume that the highly concentrated solutions are metastable and over time the compound agglomerates and precipitates from the solution, which leads to a lower measured concentration. This could mean that the effectively leached amounts of wood preservatives from the reference samples are even higher than measured by GC-MS. The amounts of tebuconazole and propiconazole released from the reference samples are, therefore, at least 100 times higher than the amounts diffusing from the wood composites, despite the lower wood-to-preservative ratio in the reference samples. At least 35% of the applied tebuconazole and 35% of the applied propiconazole leached out of the resin-free wood reference samples.

As mentioned earlier, no permethrin could be detected in the wood composites. However, in both types of reference samples a significant amount of permethrin is detected. In this case, one can assume that the amount of permethrin released by the wood composites has to be below 0.6 µg/mL (limit of detection), which corresponds to less than 0.01% of the permethrin applied and is three orders of magnitude lower than found for the reference samples. This means that the resin matrix apparently binds permethrin either completely or to a very high extent, preventing its release efficiently, whereas a permethrin concentration of at least 32 µg/mL was detected in the reference wood samples, which corresponds to 21% of the applied permethrin leached out of the resin-free wood reference samples.

#### 3.2.3. GC-MS Study of Resin-Free Reference Wood Millings

**Figure 4:**
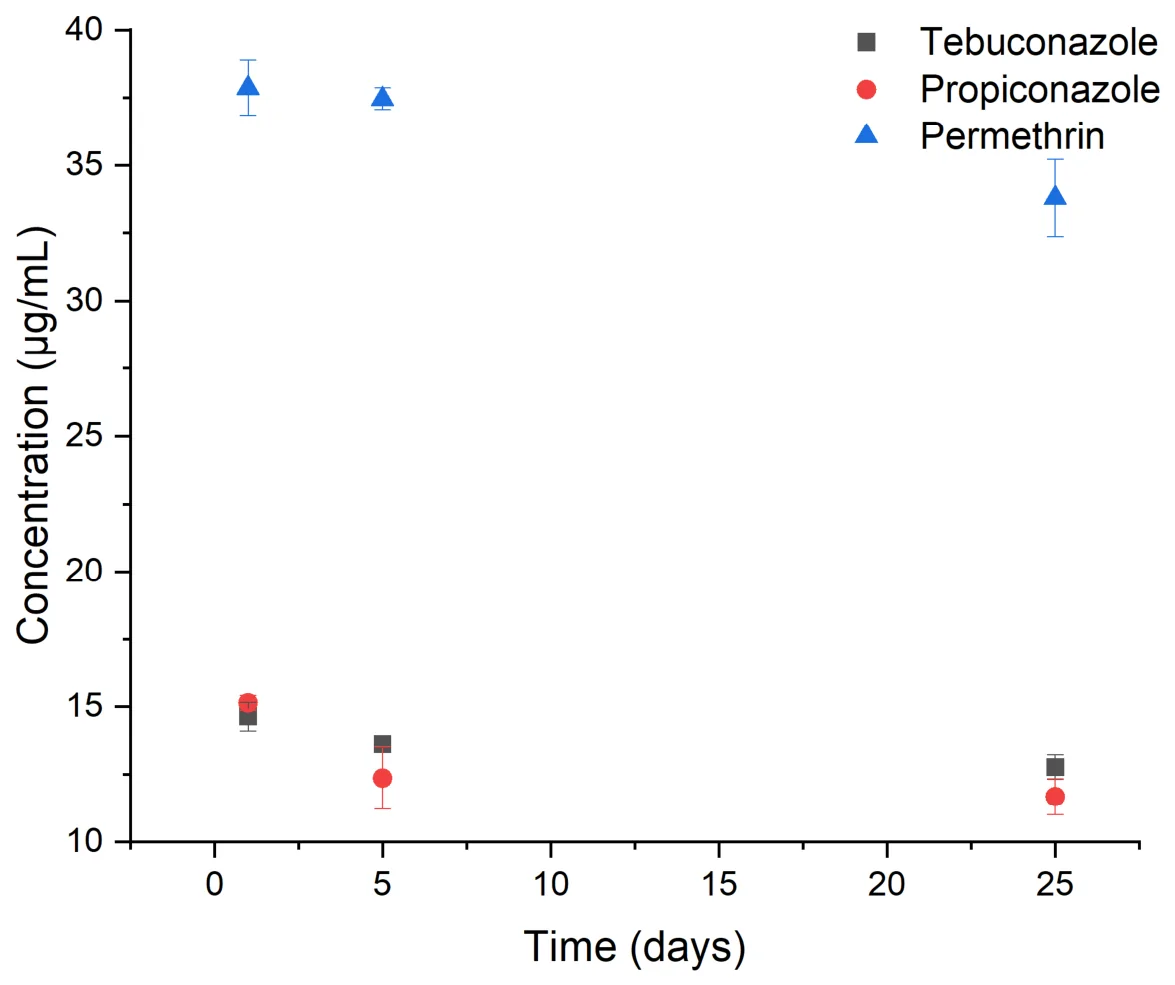
Leaching of propiconazole and tebuconazole from wood reference millings into water: the concentrations of propiconazole (red), tebuconazole (black), and permethrin (blue) measured in the water over time, as determined by GC-MS. The sampling was done after 1 day, 5 days, and 25 days.

A similar picture is seen with the reference wood millings: the amounts of tebuconazole, propiconazole, and permethrin measured in the water are well above the concentrations released from the wood composites (see Figure 4).

The surprisingly high concentration of permethrin (approximately 38 µg/mL after 1 day) is even more pronounced in this case, and the difference in the concentration of permethrin compared to propiconazole and tebuconazole (concentrations of 14–15 µg/mL) is also higher. Interactions of the substances tebuconazole and propiconazole with the wood chips seem to play a role. The more polar substances propiconazole and tebuconazole may undergo stronger interactions with the wood chips than the more apolar permethrin. However, this effect is clearly weaker than the encapsulation effect of the resin matrix on the substances in the wood composites. It is more pronounced in the wood millings than in the reference wood pucks because the surface-to-volume ratio of the chips is larger. In the wood milling reference samples, the concentrations of permethrin, tebuconazole, and propiconazole also decrease slightly over time, which is presumably due to the precipitation of the compounds in water over time. Thus, it is possible that the actual amounts of released substances are higher than those detected by GC-MS.

In the case of the wood chips, the initial concentrations of the wood preservatives change only marginally over time. One possible reason could be that due to the higher surface-to-volume ratio of the wood chips, more of the emulsifying substances present in the formulation (Korasit NG10) can diffuse into the water, thereby stabilising permethrin. While these experiments demonstrate that the resin is crucial to encapsulating the wood preservatives effectively, the experiments cannot fully quantify and differentiate the contribution of the resin from the adhesion between wood surface and wood preservatives and the influence of additives.

## 4. Discussion

UV-Vis spectroscopy of wood composites treated with dyes was used to provide a proof-of-principle: within a wood composite, small molecules can be encapsulated and retained to a relatively high degree. Two dyes were chosen that differ greatly in their water solubility: Nile red has a very low water solubility (solubility less than 1 mg/mL at 20 °C) and uranine has a very high water solubility (solubility more than 100 g/L at 20 °C). The degree to which these molecules can be encapsulated varied according to the dye used: no Nile red could be detected in the water in which the Nile-red-treated wood composite had been stored in for eleven days. It appears that at most trace amounts of the dye leach into the water. Small quantities of uranine could be detected in the water that the uranine-treated wood composites had been stored in for eleven days. The detected quantities vary between 2% and 4% of the initially used dye. The slight variations obtained for the different resin compositions suggest that the polymer network of the resin may have an influence on the leaching of the small molecules trapped within. These results were taken into account when choosing the resin composition for further GC-MS experiments investigating the leaching of small molecules from wood composites. 

The underlying factors governing leaching differences between the small molecules are not entirely clear. As expected, the water solubility plays a crucial role in the leaching behaviour of the compounds but is not solely responsible for the differences in leaching, as evidenced by the results of the GC-MS study. 

In the GC-MS study, wood composites were produced that had been pre-treated with a wood preservative formulation containing propiconazole, tebuconazole, and permethrin and subjected to a leaching experiment. The leaching of the wood preservatives into the water was followed over time. Here, too, differences in leaching were found between the three molecules. While the theory that water solubility plays a major factor in the leaching behaviour of molecules seems to be substantiated by the fact that permethrin, a molecule with very low water solubility (less than 0.2 mg/mL), could not be detected in the water used for the leaching experiment, the leaching results found for propiconazole and tebuconazole are not consistent with this hypothesis. Both tebuconazole and propiconazole leached into the water; however, while propiconazole has a higher solubility in water (100–150 mg/L) than tebuconazole (32–36 mg/mL), less propiconazole had leached into the water than tebuconazole. At all sampling intervals, the concentration of tebuconazole found in the water was roughly double the measured concentration of propiconazole. One explanation could be that propiconazole has a higher hydrodynamic volume than tebuconazole. Reynier et al. have shown that flexibility, shape, and size are crucial for the migration of substances within polymer matrices. Since they examined migration within thermoplastics, while leaching from a crosslinked thermoset is studied here, this comparison remains qualitative, but it can be expected that the underlying mechanisms might still apply [31,32]. 

A comparison with the resin-free reference samples strongly suggests that the resin plays a major role in encapsulating the wood preservatives. Without the resin, the concentrations of tebuconazole, propiconazole, and permethrin found in water after leaching are significantly higher than in the case of the wood composites. Interestingly, the amount of permethrin, tebuconazole, and propiconazole that leached from the resin-free reference samples differed between resin-free wood samples and resin-free wood millings. Specifically, tebuconazole and propiconazole exhibit weaker leaching from the wood millings. We hypothesise that these compounds undergo interactions with the wood that reduce their leaching into water. 

The results show that the wood composites are capable of encapsulating large amounts of the small molecules. The conducted experiments showed that it is possible to encapsulate wood preservatives or other potentially hazardous substances contained in wood using a resin matrix. The extent of encapsulation varies according to the substance and is most likely also dependent on the resin composition. In the case of the active ingredients of the wood preservative, more than 99% of the wood preservatives remain in the wood composites and do not leach into the water. This is an important proof-of-principle and, to the best of our knowledge, has not been studied for organic wood preservatives before. This is a first step towards reusing contaminated wood as reinforcement material in composites, thereby reducing the amount of fresh wood required for the fabrication of wood composites and expanding the lifecycle of the wood. With regard to the safe use of preservative-treated wood for wood composites, further research is required to assess the behaviour outside of the laboratory, namely, how the leaching behaviour from wood composites would be influenced by aging processes, such as weathering and mechanical and biological degradation. While only small amounts of uranine, propiconazole, and tebuconazole leached into water, these results may differ for other preservatives, making it necessary to study the predictability of preservative leaching based on their properties. As the amount of preservative leached is dependent on the molecule and the resin composition, further studies should aim at tailoring the resin composition to the specific substance that needs to be encapsulated. Furthermore, this work focuses on the retention of preservatives applied to freshly impregnated virgin spruce, which may differ from the leaching behaviour of composites produced from aged, preservative-treated wood. Since a fraction of the actives is depleted during service life, the total leachable load of aged wood is expected to be lower. This hypothesis, however, remains to be verified experimentally.

## 5. Conclusions

This study demonstrated that wood composites are capable of encapsulating and retaining small molecules to a high degree, as evidenced by the low leaching of two fluorescent dyes (Nile red and uranine) and three organic wood preservatives (propiconazole, tebuconazole, and permethrin) into water. UV-Vis spectroscopy provided a proof-of-principle, since the highly water-soluble dye uranine leached only 2–4% of its initial amount, while the almost insoluble Nile red remained undetectable in the aqueous phase. The GC-MS experiments confirmed that more than 99% of all three preservatives remained within the composite after 26 days of water storage. Comparing these results with reference materials (i.e., treated wood pucks and wood millings) made clear that the resin matrix was crucial for this retention. The resin-free wood references released significantly higher amounts of the investigated preservatives. Interestingly, the leaching behaviour did not strictly correlate with water solubility, pointing to additional factors, such as molecular size, polymer network interactions, and specific wood–molecule interactions. To our knowledge, this work provides a first demonstration that wood contaminated with organic wood preservatives can be effectively encapsulated in a composite, offering a promising route for reusing such wood and extending its lifecycle. However, before practical application, further research is needed to evaluate the long-term stability of the encapsulation under environmental conditions (e.g., weathering and mechanical and biological degradation), to investigate the influence of different resin compositions on retention efficiency, and to verify the leaching behaviour of composites made from aged, partially depleted preservative-treated wood. Ultimately, these findings lay the groundwork for designing composite materials that effectively immobilise hazardous substances, contributing to a more sustainable and safer use of wood resources.

## Figures and Tables

**Table 1:** Compositions of the resins used for the UV-Vis spectroscopy study of the leaching of dyes uranine and Nile red from wood composites. The quantities are expressed in wt%.

Formulation	Resin (wt%)	S (wt%)	ACMO (wt%)	DVB (wt%)	I (wt%)	Additive (wt%)
R	99				1	
1	79.2	19.8			1	
2	79.2		19.8		1	
3	79.2			19.8	1	
4	78.8	19.7			1	0.5 (M1)
5	78.8	19.7			1	0.5 (M2)

**Table 2:** Masses of wood and wood preservative used in the preparation of resin-free reference samples, including maximum theoretical concentration of wood preservative ingredients that could be found in a GC-MS sample.

Sample	m (Wood)	m (Korasit NG10)	Theoretical Max. Concentration of Tebuconazole (T), Propiconazole (PR), and Permethrin (PE) in mg/mL
WP1	4263 mg	2852 mg	T: 0.085; PR: 0.085; PE: 0.142
WP2	4323 mg	2884 mg	T: 0.086; PR: 0.086; PE: 0.144
WP3	4290 mg	2866 mg	T: 0.086; PR: 0.086; PE: 0.143
Millings	7500 mg	5625 mg	T: 0.084; PR: 0.084; PE: 0.141

**Table 3:** Quantification of the leaching of uranine from different wood composites into water, as determined by UV-Vis spectroscopy.

Resin Formulation	Concentration of Uranine (µg/mL)	Leaching (%)
R	0.886 ± 0.004	2.95
1	0.808 ± 0.012	2.69
2	0.998 ± 0.003	3.33
3	1.176 ± 0.009	3.92
4	0.654 ± 0.013	2.18
5	0.719 ± 0.066	2.40

## Data Availability

The original contributions presented in this study are included in the article. Further inquiries can be directed to the corresponding authors.

## Abbreviations

The following abbreviations are used in this manuscript:

WPC	Wood–plastic composites
FAO	Food and Agriculture Organization of the United Nations
IPBC	3-iodo-2-propinyl butylcarbamate
UV-Vis	Ultraviolet-visible
GC-MS	Gas chromatography–mass spectrometry
DVB	Divinylbenzene
ACMO	4-Acryloylmorpholine
MeCN	Acetonitrile
S	Styrene
T	Tebuconazole
PZ	Propiconazole
PM	Permethrin
